# Implementation strategy mapping methods to improve autism intervention use in community settings: a study protocol

**DOI:** 10.1186/s43058-022-00339-6

**Published:** 2022-08-18

**Authors:** Aksheya Sridhar, Amy Drahota, Jessica E. Tschida

**Affiliations:** Department of Psychology, Michigan State University, East Lansing, MI USA

**Keywords:** Implementation strategies, Community mental health, Autism spectrum disorders

## Abstract

**Background:**

Implementation strategies are purported to facilitate adoption and use of evidence-based practices (EBPs) across settings. The use of tailored implementation strategies may be particularly effective, as they are selected with the explicit purpose of addressing setting-specific implementation determinants. However, methods to select and tailor implementation strategies, including in community settings, remain understudied. This project will identify and describe implementation strategy mapping methods (ISMMs) from extant peer-reviewed literature and pilot test a method to match implementation strategies with determinants in low-resourced community mental health (CMH) agencies that deliver services to children on the autism spectrum.

**Methods:**

Aim 1: A scoping review, following PRISMA guidelines, will be conducted to identify implementation strategy mapping methods (ISMMs) utilized in child mental health settings. Data extraction will identify and describe each ISMM, including identifying methodological and procedural steps, analyzing the frequency of ISMM use, and identifying outcomes measured in eligible ISMM studies.

Aim 2: Using scoping review findings, select and pilot test one ISMM within five community mental health agencies in Michigan that provide services to autistic children. We will recruit five directors/agency leaders, supervisors, and direct providers at each of the eligible agencies (expected *N* = 25). A sequential explanatory (QUAN➔ QUAL) mixed methods design will be used. Participants will complete a demographics and client survey, as well as a needs assessment to identify implementation determinants. The impact of the ISMM on organizational readiness for change (from pre- to post-ISMM), as well as implementation outcomes of the ISMM (feasibility, acceptability, appropriateness, usability), will be examined. Semi-structured interviews will elicit stakeholder perspectives on the mapping method.

**Discussion:**

The current project aims to advance our knowledge of methods for selecting, tailoring, and mapping implementation strategies to address context-specific determinants to implementation. Additionally, this project will contribute to growing science found at the intersection of implementation science and autism research by utilizing the implementation determinants framework, the CFIR, to guide data collection, analysis, and interpretation of findings. Finally, these findings may support future EBP implementation efforts within low-resourced communities, with the ultimate goal of increasing equity in access to EBPs for autistic children.

Contributions to the literature

- This study will explore methods to select and tailor implementation strategies to specifically address implementation barriers and enhance implementation facilitators
- Methods that have been previously used within the context of child mental health service delivery will be identified
- One method will be tested in the context of community agencies in Michigan that provide services to autistic children from low-income backgrounds
- Findings will advance our understanding of effective methods to select and tailor implementation strategies so that they are best suited to the settings in which implementation is occurring

## Background

Implementation science seeks to increase the uptake and utilization of evidence-based practices (EBPs) in community-based usual care settings, in an effort to reduce the gap that exists between EBP use in research and practice settings [1]. However, these efforts are not without their challenges, including a lack of systematic and effective implementation processes in community-based usual care settings to facilitate the adoption and implementation of EBPs [2]. Furthermore, while similar challenges may be present across different community-based organizations, each organization faces context-specific implementation barriers and facilitators (i.e., determinants) that are unique to their setting. *Implementation strategies* are defined as a potentially effective way to increase the adoption, utilization, and sustainment of EBPs across settings [3–5]. Furthermore, the use of implementation strategies that are tailored to the organizations in which they will be utilized may be particularly effective in efforts to address implementation determinants and enhance EBP use [6].

A large number of implementation strategies have been identified in extant literature. For example, the Expert Recommendations for Implementing Change (ERIC; [7]) is a commonly used list comprised of 73 distinct implementation strategies. Of importance, it is unknown which of these strategies are most effective within specific service settings or which strategy may address specific organizational barriers (e.g., lack of funding, limited provider training) to EBP implementation, especially within low-resource community service settings [8]. Moreover, although researchers have hypothesized that tailored implementation strategies may be particularly effective for addressing determinants, there is a lack of consensus and guidance in the literature regarding systematic methods for selecting and tailoring implementation strategies for different contexts, including how to map these strategies onto identified implementation barriers and facilitators. Indeed, “enhancing methods for designing and tailoring implementation strategies” has been identified as a high priority aim within the field of implementation science [4].

Implementation researchers have put forward some methods to select and tailor implementation strategies. For example, Powell and colleagues outlined numerous challenges with selecting and tailoring implementation strategies and proposed four methods for matching implementation strategies to determinants: concept mapping, group model building, conjoint analysis, and intervention mapping. These methods have all begun to be utilized within the context of behavioral health service delivery [9]. Other methods include intervention/implementation mapping, which has also been utilized within the healthcare setting [10], as well as the CFIR-ERIC Implementation Strategy Mapping Tool [6] which has been utilized within adult mental health care delivery settings [11]. However, it is currently unknown which specific implementation strategy or set of strategies are likely to be most impactful and feasible in various settings, including in community mental health settings.

In Michigan, community mental health (CMH) agencies are a key service system for providing interventions to autistic children. Moreover, these systems are essential in providing services to autistic children enrolled in the Michigan Medicaid Autism Benefit, who have a household income at or below 133% of the federal poverty level [12]. Although these systems are vital in providing services to autistic children experiencing socioeconomic disadvantage, research indicates that CMH agencies utilize EBPs developed for this population (ASD-EBPs) at a low frequency and with varied intensity [12, 13]. Given the limited use of ASD-EBPs within Michigan CMH agencies, autistic children experiencing socioeconomic disadvantage may receive interventions at a significantly lower rate compared to other children from less disadvantaged backgrounds. Therefore, there is a critical need to investigate methods to systematically and equitably increase the use and delivery of ASD-EBPs within CMH agencies providing services to autistic children from low-resourced communities and experiencing socioeconomic disadvantage [14].

In addition, there is a strong need for the use and delivery of ASD-EBPs for autistic children given the growing prevalence of ASD and potential for associated challenges. ASD is estimated to impact 1.8% of the US population and diagnoses are estimated to be given to 1 in 44 children [15]. ASD is a pervasive neurodevelopmental disorder characterized by social communication differences and the presence of repetitive and/or restricted behaviors [16]. Children on the autism spectrum may be more likely to have difficulty in areas such as social communication, adaptive behavior, and executive functioning compared to neurotypical children, and face considerable systemic barriers to inclusion [17, 18]. Moreover, these difficulties may be exacerbated for autistic children with marginalized identities or experiencing marginalized circumstances (e.g., socioeconomic disadvantage) given the lower rates at which they receive EBPs demonstrated to improve outcomes [13, 19, 20].

Numerous ASD-EBPs have been identified and found to improve both core and co-occurring symptoms (e.g., mental health concerns) for autistic children on average [21]. Naturalistic Developmental Behavioral Interventions (NDBIs) are one category of ASD-EBPs that involve in-session practice that focuses on skill development within a natural environment and during everyday routine activities [21]. Within this ASD-EBP category, Project ImPACT is a parent-mediated NDBI that coaches parents on how to improve their autistic child’s social engagement, social communication, imitation, and play skills within everyday activities [21, 22]. Notable benefits to utilizing NDBIs and parent-training models like Project ImPACT include increased intervention dosage as parents can deliver intervention techniques throughout the day and within varied situations that allows for greater generalization of skills [23]. Further, research evaluating Project ImPACT in both research and community/usual-care settings indicates that this intervention significantly improves communication skills (e.g., greater language acquisition) as well as lowers parental stress [24, 25]. Considering the multiple benefits (e.g., developed in collaboration with autism stakeholders) and demonstrated outcomes of Project ImPACT, it appears a promising fit for use within service systems, like CMH agencies, that have a critical need for feasible and effective EBPs for autistic. However, methods to select and tailor implementation strategies to most effectively implement Project ImpACT within CMH agencies remain unclear.

The current study aims to (a) identify methods that may be used to select and tailor implementation strategies to unique contexts, and (b) evaluate implementation outcomes and organizational impact of one implementation strategy mapping method (ISMM) within the context of CMH agencies. Specifically, this project will focus on implementation efforts within CMH agencies in Michigan that are interested in utilizing Project ImPACT with their autistic clients enrolled in Medicaid benefits. Exploring methods to systematically tailor the implementation process and increase utilization of Project ImPACT within CMH agencies may support increased service equity and ultimately improve patient outcomes for autistic children experiencing socioeconomic disadvantage. The work in the funded project will proceed in two phases with two corresponding specific aims:

- *Aim 1:* Conduct a scoping review of implementation strategy mapping methods (ISMMs), or methods to select, tailor, and map implementation strategies, to address unique determinants within the context of child mental health service delivery settings.
- *Aim 2:* Pilot test the use of one ISMM within Michigan CMH agencies providing services to autistic youth whose services are funded by the Medicaid Autism Benefit. Specifically, aim 2 will examine whether the ISMM increases organizational readiness to change from pre- to post-ISMM, and will evaluate implementation outcomes (i.e., perceived feasibility, acceptability, and appropriateness; usability) of the ISMM for selecting and tailoring implementation strategies.

## Method

### Aim 1: conduct a scoping review of implementation strategy mapping methods (ISMMs), or methods to select, tailor, and map implementation strategies to address unique determinants within child mental health service delivery settings

#### Design

The scoping review will facilitate understanding of the extent to which ISMMs have been studied within the context of child mental health service delivery settings, as well as to synthesize the evidence related to ISMMs. Given the large number of discrete implementation strategies, these findings are expected to advance our understanding of how to select strategies that best fit an organization and inform future tailored EBP implementation efforts. Findings will inform which ISMM strategy will be pilot tested during phase 2 (Aim 2).

#### Materials

Covidence software will be utilized to facilitate the title and abstract and full-text review phases of the scoping review. MAXQDA, a qualitative analysis software, will be utilized during data charting.

#### Processes

The scoping review will follow PRISMA guidelines and procedures [26, 27]. The literature search will include three steps to (1) identify common methods, (2) search the literature for identified methods based on the first step, and (3) review references from the included papers to identify additional literature. For details on search terms and databases utilized, see Table 1. Articles identified in the literature review will be screened using a title and abstract review. Finally, articles determined to be eligible following the title and abstract review will be evaluated in the full-text review (Table 2).

**Table 1:** Scoping review search terms and databases

Search terms/description	Databases
(“implementation strategy” or “implementation strategies”) AND (“child” OR “pediatric” OR “children”) AND (“mental health service” OR “evidence-based practice” OR “evidence based intervention” or “mental health treatment”)	PsycInfo
	Social Services Abstracts
	PubMed
(“concept mapping” OR “conjoint analysis” OR “group model building” OR “intervention mapping”) AND (“implementation strategy” OR “implementation strategies”) AND (“child” OR “pediatric” OR children) AND (“mental health service” OR “evidence-based practice” OR “evidence based intervention” or “mental health treatment”)	PsycInfo
	Social Services Abstracts
	PubMed
Review references in papers identified via searches 1 and 2 to identify additional relevant literature	N/A

**Table 2:** Scoping review eligibility criteria

Inclusion criteria	Exclusion criteria
Title and abstract review	
- Discusses selecting/tailoring/mapping implementation strategies to address implementation determinants - Describes a method for selecting/tailoring/ mapping implementation strategies - Providers work with child populations (< 18) - Implementing mental health practices/interventions	- Article not in English - Providers who work exclusively with adult populations (> 18 years) [If providers work with a range (e.g., 16–25) that includes individuals under 18 years old, *include*] - Non mental-health practices/interventions
Full-text review	
- Includes description of a strategy/method (e.g., concept mapping, intervention mapping) for tailoring implementation strategies to identified determinant - Delivery of MH-EBPs to child populations	- Article not in English Sample details: - Providers who work exclusively with adult populations (18 +) [If providers work with a range (e.g., 16–25) that includes individuals under 18 years old, *include*] - Non mental-health practices/interventions - Article does not provide information on the population of focus ISMM details: - Article does not mention selecting/tailoring/mapping implementation strategies - Article does not describe the method for selecting/tailoring/mapping implementation strategies

#### Analyses plan

Data extraction will be completed for all articles included following the full-text review. Data will be examined by (a) evaluating the frequency with which each strategy is utilized, (b) conducting content analyses to describe the methods utilized in the literature, and (c) identifying common outcomes measured. Additional data about the rigor of the evidence overall for each method and feasibility data will be gathered.

### Aim 2: pilot test the use of one ISMM within Michigan CMH agencies providing services to autistic youth whose services are funded by the Michigan Medicaid Autism Benefit

Specifically, aim two will examine whether the ISMM increases organizational readiness to change from pre- to post-ISMM, and will evaluate implementation outcomes of the ISMM for selecting and tailoring implementation strategies, including perceived feasibility, acceptability, appropriateness, and usability.

#### Design

**Fig. 1:**
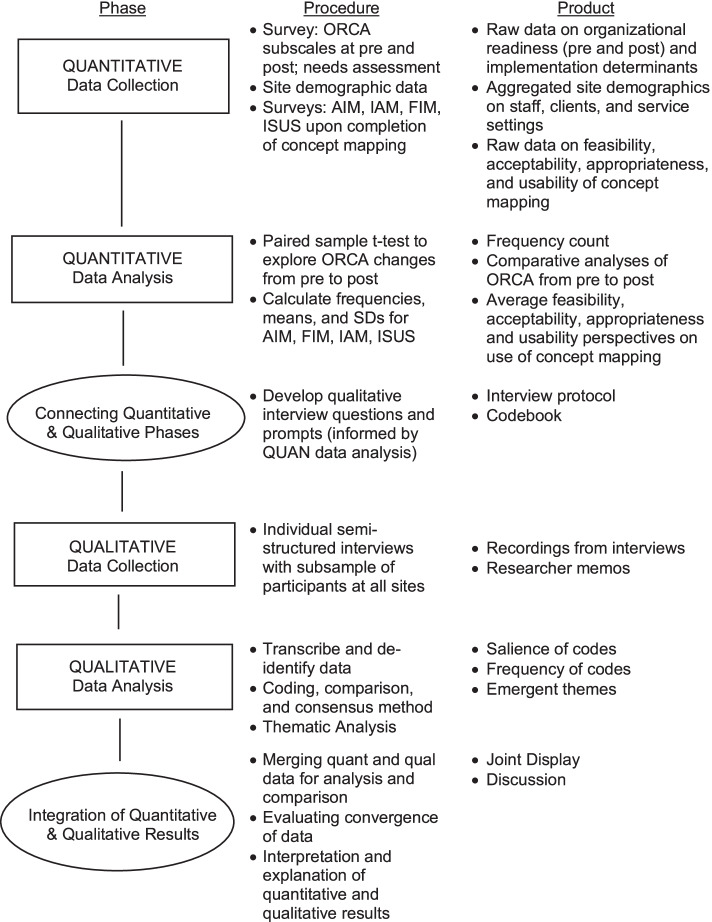
Study design

A sequential explanatory (QUAN➔QUAL) mixed methods design (Fig. 1) will be utilized for Aim 2. We will evaluate the feasibility, acceptability, appropriateness, and usability of the ISMM for each participating organization; examine stakeholder perspectives on their organization’s readiness for change; and further explore stakeholder perspectives regarding the ISMM using semi-structured interviews.

#### Setting

Purposeful sampling will be utilized to recruit five community-mental health (CMH) agencies in Michigan. Agencies interested in participating will attend a recruitment meeting with the Principal Investigator to review study details and benefits. Agencies will be eligible if they (a) provide services to children on the autism spectrum who are enrolled in Medicaid benefits, (b) identify a need for implementing Project ImPACT within their agency, and (c) endorse an interest in utilizing systematic implementation strategies to facilitate this process.

#### Participants

We will recruit five directors/agency leaders, supervisors, and direct providers at each of the eligible agencies (expected *N* = 25). Participants will be recruited during the agency recruitment meeting and will be asked to complete an online screening form to determine eligibility, if they are interested in participating. Directors/agency leaders will be eligible if they fulfill the role of director or leading decision-maker regarding interventions provided within their agency. At least 1 director/agency leader will be required to participate. Supervisors and direct providers will be eligible if they provide or oversee staff who provide interventions to autistic children who receive their services via the Michigan Medicaid Autism Benefit. Agency staff will be ineligible if they do not read or speak in English.

#### Materials

Aim 2 materials include quantitative measures (described below), qualitative interviews (described below), the ERIC list of implementation strategies, and MAXQDA qualitative coding software. Additionally, any software associated with the selected ISMM may be utilized (e.g., GroupWisdom software for concept mapping).

#### Quantitative measures

##### Demographic and client survey

A questionnaire will be administered to collect provider and organizational demographics as well as information about the clientele receiving services through the CMH setting prior to the first phase of concept mapping.

##### Needs’ assessment

A strength and needs’ assessment based on the Consolidated Framework for Implementation Research (CFIR; [28]). The CFIR is a commonly used determinant framework that facilitates understanding of an implementation context through the identification of implementation barriers and facilitators. This framework includes five domains: intervention/innovation characteristics, outer setting, inner setting, individual characteristics, and implementation process.

##### Readiness for change

Three measures will be utilized to measure readiness for change prior to, and upon completion of, the ISMM. The Organization Readiness for Implementing Change (ORIC; [29]), Organizational Readiness for Change Assessment (ORCA) context scale [30], and Organizational Readiness for Change (ORC; [31]) will be used to elicit participant perspectives regarding their organization’s motivation and capacity to support and facilitate the use of new interventions, such as Project ImPACT, within their organization.

##### Implementation outcomes

Participants will be asked to complete measures evaluating the implementation outcomes of the ISMM. The first three surveys evaluating feasibility, acceptability, and appropriateness are comprised of 4 items, utilizing a 4-point Likert scale (1—“completely disagree” to 4— “completely agree”). These measures have robust psychometric properties, including discriminant content validity and test–retest reliability, and have been utilized across contexts [32]. The last survey is comprised of 10 items and uses a 5-point scale (strongly disagree to strongly agree; [33]).

**Feasibility of Intervention (FIM)**

The FIM will examine participant perspectives on “the extent to which a new… innovation, can be successfully used or carried out within a given agency or setting” (e.g., “[ISMM] seems easy to use”).

**Acceptability of Intervention Measure (AIM)**

The AIM will measure “the perception among implementation stakeholders that a given treatment, service, practice, or innovation is agreeable, palatable, or satisfactory” (e.g., “[ISMM] meets my approval”).

**Intervention Appropriateness Measure (IAM)**

The IAM will examine “perceived fit, relevance, or compatibility of the innovation for a given practice setting, provider, or consumer; and/or perceived fit of the innovation to address a particular issue or problem” (e.g., “[ISMM] seems fitting”).

**Implementation Strategy Usability Scale (ISUS)**

The ISUS will examine the perceived usability—“the extent to which an intervention can be used by specified users to achieve specified goals with effectiveness, efficiency, and satisfaction” of the concept mapping method. (e.g., “I think I would like to use [ISMM] frequently”).

#### Qualitative interview

##### Semi-structured individual interviews

The interview protocol will be developed following quantitative data collection and analysis. The interview will further explore staff perspectives on the impact of the ISMM on the organization’s readiness to change, implementation outcomes related to the ISMM, and suggestions to improve the ISMM. Interview questions will be developed to align with constructs from the CFIR. Participants will be asked about perspectives on intervention (i.e., ISMM) characteristics, outer and inner settings factors, and individual (i.e., staff) characteristics that influenced the perceived feasibility, acceptability, and appropriateness of the ISMM as well as how these factors influenced perceived organizational readiness for change.

#### Processes

Aim 1 findings will inform the selection of one ISMM that is identified as (1) being evidence-based and feasible for use and (2) an approach that includes stakeholder engagement in the process.

Regardless of the ISMM selected after completing the scoping review, measures and data analysis will remain the same. Additionally, initial research procedures are similar across ISMMs, and steps to map implementation strategies will be adjusted according to the ISMM selected. All ISMM methods begin with a process for identifying implementation barriers and facilitators. Aim 2 of this study will utilize a strength and needs’ assessment to identify and prioritize implementation barriers and facilitators to implementing Project ImPACT within each agency. Next, participants at each agency will be guided to use the Expert Recommendations for Implementing Change (ERIC; [7]) list of implementation strategies. Participants will use the ERIC to select implementation strategies they believe will address identified determinants (e.g., “access new funding” to address limited funding). They will also be provided the opportunity to generate any additional implementation strategies they believe may be relevant. Next, participants will sort and rank strategies in order to prioritize implementation strategies that may be most feasible and useful for their setting, as well as tailor strategies as needed. Procedures to tailor or adapt strategies will depend on the ISMM selected.

#### Analyses plan

##### Quantitative analysis plan

The quantitative data collected from questionnaires will be analyzed in three phases. First, analyses will focus on detecting and correcting potential data errors, describing properties, and evaluating the quality of the data. Descriptive analyses will be completed (i.e., means, frequencies, distributions) to report demographic data. Given the limited sample size, paired sample *t*-tests will be conducted to test for changes in perceptions as reported on the ORCA, ORIC, and ORC between pre-and post-ISMM at each agency. Finally, mean responses to the AIM, IAM, FIM, and ISUS will be aggregated by agency after completing the ISMM process to examine the implementation outcomes associated with the ISMM.

##### Qualitative analysis plan

Interview data will first be transcribed and verified by the research team. We will utilize thematic analysis [34] to analyze the qualitative interviews. Two independent coders will collaboratively develop a coding schema to explore concepts related to ISMM implementation outcomes, impact on organizational readiness for change, and overall perspectives regarding the ISMM, in alignment with CFIR constructs across all 5 domains. Emergent codes will also be identified when relevant. Next, the frequency and saliency of each code will be determined, and codes will be grouped into broader categories. The final step will be to identify overarching themes that summarize the qualitative data. Consensus coding procedures will be utilized throughout the process to address coding discrepancies. All data will be analyzed with MAXQDA software. We will adhere to the Standards for Reporting Qualitative Research [35] in order to ensure transparency and accuracy throughout qualitative data collection, analyses, and reporting.

##### Integration of QUAN and QUAL data strands

Qualitative data will be quantized to examine the frequency of each code across interview transcripts and to facilitate assessment of the saliency of each code. Both the quantitative and qualitative data strands will be analyzed independently first. Next, data strands will be merged in a joint display (i.e., side by side comparison table; [36]) in order to integrate findings from both quantitative and qualitative data, to understand where participant’s perspectives may converge or diverge, and to contextualize the quantitative findings. For example, quantitative data on the average acceptability of the ISMM will be explored at each organization and then further explored through utilization of the qualitative findings, when contrasting and comparing these findings in the joint display. Overall, merging the quantitative and qualitative data strands will allow for a deeper understanding of the specific components that participants found acceptable, factors influencing their perspectives on acceptability, and barriers and facilitators to acceptability.

## Discussion

### Innovation and impact

Overall, the current project, which includes a scoping review and pre-implementation study, aims to advance our knowledge of effective methods for selecting, tailoring, and mapping implementation strategies to context-specific implementation determinants. Specifically, this project will provide the first scoping review of methods to select, tailor, and map implementation strategies to address unique determinants within a given setting (Aim 1). Given the large number of discrete implementation strategies, these findings will advance our knowledge of how to select strategies that best fit an organization and inform future tailored EBP implementation efforts. Secondly, this project will be the first to evaluate the use of an ISMM within CMH agencies that provide services to autistic children experiencing socioeconomic disadvantage. The pre-implementation study (Aim 2) will substantially advance our knowledge of effective strategies for selecting, tailoring, and mapping implementation strategies to combat barriers and leverage facilitators that are unique to Michigan CMH agencies. Findings will support future ASD-EBP implementation efforts within low-resourced communities, with the ultimate goal of increasing equity in access to EBPs for this population. Finally, this project will contribute to growing science found at the intersection of implementation science and autism research by utilizing an implementation determinants framework, the CFIR, to evaluate the use of an ISMM within the context of CMH agencies providing services to autistic youth from low-resourced communities. While this framework has been previously utilized or is currently being tested in studies exploring implementation strategy mapping, it has not yet been used to explicitly guide data collection and analysis. Therefore, this study will utilize a mixed-methods research design to incorporate the CFIR as a guiding framework to understand stakeholder perspectives related to the use of the ISMM within CMH agencies providing services to autistic youth.

### Considerations and limitations

While this innovative line of study has potential for ultimately increasing equitable access to ASD-EBPs for autistic youth and their families through improved implementation processes, specific project limitations exist and must be considered. The scoping review (Aim 1) may not yield only one feasible and effective ISMM (e.g., equivocal outcomes may be found for 2 or more ISMMs). Studies of ISMMs are nascent; thus, there may be a paucity of existing literature evaluating ISMM strategies. Further, article eligibility/ineligibility criteria may reduce the number of articles included in the scoping review. If the scoping review does not yield one obvious ISMM to utilize in Aim 2, study personnel will discuss advantages and disadvantages of the identified ISMMs, consult with implementation science and community engagement experts, and determine one specific ISMM to be utilized in the pre-implementation study.

Additionally, because the goal of Aim 2 is to conduct foundational pilot research on the acceptability, feasibility, appropriateness, and usability of an ISMM, other implementation outcomes, such as adoption, fidelity, costs, and sustainability, will not be evaluated. While important constructs within implementation science, recent guidance suggests the need to conduct feasibility and pilot studies on implementation strategies, such as ISMMs, in order to identify potential revisions to the strategy, refine the research design and procedures for future implementation studies, or test the preliminary effects of the strategy on relevant implementation, service and individual-level outcomes [37, 38]. As stated by Proctor and colleagues [5], “the study of implementation strategies should be approached in a similar fashion as evidence-based interventions, for strategies are in fact a type of intervention” (p. 3). Thus, implementation strategies, in their own right, should be empirically tested prior to broader use to ensure that only evidence-based implementation strategies are being disseminated and utilized in usual care settings [4]. Further, this study will include a small number of CMH agencies in Aim 2 study procedures. As a result, the results of the pilot study may not be generalizable to broader systems, geographical locations, or populations. However, the goals of Aim 2 are to gather feasibility data-related implementation strategy procedures and study methods as well as assess potential implementation strategy effects to the use of ISMM within this common service system for autistic youth (e.g., prioritizing external validity) [5, 37, 39].

## Conclusion

Overall, the purpose of this study is twofold. First, this study seeks to identify methods that have been used to select, tailor, and map implementation strategies to context-specific determinants within child mental health service delivery settings. Second, this study seeks to pilot test one method within Michigan-based community mental health agencies providing services to autistic children experiencing socioeconomic disadvantage. This pre-implementation study seeks to advance our knowledge of effective strategies for selecting, tailoring, and mapping implementation strategies, in order to combat barriers to implementation that are unique to CMH agencies, as well as enhance facilitators within these settings. These findings will support future studies that test the effectiveness of tailored implementation strategies to increase ASD-EBP use in CMH agencies, with the ultimate goal of improving access to services for autistic youth.

## Data Availability

The datasets used and/or analyzed during the current study will be available from the corresponding author on reasonable request.

## Abbreviations

ASD: Autism spectrum disorder
EBP: Evidence-based practice
CMH: Community mental health
ISMM: Implementation strategy mapping methods
ERIC: Expert Recommendations for Implementing Change
CFIR: Consolidated Framework for Implementation Research
ORCA: Organizational Readiness for Change Assessment
ORC: Organizational Readiness for Change
ORIC: Organizational Readiness for Implementing Change
IAM: Appropriateness of Intervention Measure
AIM: Acceptability of Intervention Measure
FIM: Feasibility of Intervention Measure
ISUS: Implementation Strategy Usability Scale

## References

[1] Green AE, Fettes DL, Aarons GA (2012). A concept mapping approach to guide and understand dissemination and implementation.

[2] Drahota A, Meza RD, Bustos TE, Sridhar A, Martinez JI, Brikho B, Stahmer AC, Aarons GA (2021). Implementation-as-usual in community-based organizations providing specialized services to individuals with autism spectrum disorder: a mixed methods study. Adm Policy Ment Health.

[3] Fixsen DL, Blase KA, Naoom SF, Wallace F (2009). Core implementation components. Res Soc Work Pract.

[4] Powell BJ, Fernandez ME, Williams NJ, Aarons GA, Beidas RS, Lewis CC (2019). Enhancing the impact of implementation strategies in healthcare: a research agenda. Front Public Health.

[5] Proctor EK, Powell BJ, McMillen JC (2013). Implementation strategies: recommendations for specifying and reporting. Implement Sci.

[6] Waltz TJ, Powell BJ, Fernández ME, Abadie B, Damschroder LJ (2019). Choosing implementation strategies to address contextual barriers: diversity in recommendations and future directions. Implement Sci.

[7] Powell BJ, Waltz TJ, Chinman MJ, Damschroder LJ, Smith JL, Matthieu MM (2015). A refined compilation of implementation strategies: results from the Expert Recommendations for Implementing Change (ERIC) project. Implement Sci.

[8] Lau R, Stevenson F, Ong BN, Dziedzic K, Treweek S, Eldridge S, Everitt H, Kennedy A, Qureshi N, Rogers A, Peacock R, Murray E (2015). Achieving change in primary care—effectiveness of strategies for improving implementation of complex interventions: Systematic review of reviews. BMJ Open.

[9] Powell BJ, Beidas RS, Lewis CC, Aarons GA, McMillen JC, Proctor EK, Mandell DS (2017). Methods to improve the selection and tailoring of implementation strategies. J Behav Health Serv Res.

[10] Fernandez ME, ten Hoor GA, van Lieshout S, Rodriguez SA, Beidas RS, Parcel G, Ruiter RAC, Markham CM, Kok G (2019). Implementation mapping: using intervention mapping to develop implementation strategies. Front Public Health.

[11] Piat M, Wainwright M, Sofouli E, Albert H, Casey R, Rivest MP, Briand C, Kasdorf S, Labonté L, LeBlanc S, O’Rourke J (2021). The CFIR Card Game: a new approach for working with implementation teams to identify challenges and strategies. Implement Sci Commun.

[12] Straiton D, Groom B, Ingersoll B (2020). Parent training for youth with autism served in community settings: a mixed-methods investigation within a community mental health system. J Autism Dev Disord.

[13] Brookman-Frazee L, Drahota A, Stadnick N, Palinkas LA (2012). Therapist survey on community mental health services for autism spectrum disorders. PsycTESTS Dataset.

[14] Boyd BA, Stahmer AC, Odom SL, Wallisch A, Matheis M (2022). It’s time to close the research to practice gap in autism: the need for implementation science. Autism.

[15] Maenner M, Shaw K, Baio J, Washington A, Patrick M, DiRienzo M (2020). Prevalence of autism spectrum disorder among children aged 8 years — autism and developmental disabilities monitoring network, 11 Sites, United States, 2016. MMWR Surveill Summ.

[16] American Psychiatric Association (2013). Diagnostic and statistical manual of mental disorders.

[17] Lai MC, Lombardo MV, Baron-Cohen S. (2014). Autism.The Lancet. 201410.1016/S0140-6736(13)61539-1

[18] Mackenzie M, Cologon K, Fenech M (2016). ‘Embracing everybody’: approaching the inclusive early childhood education of a child labelled with autism from a social relational understanding of disability. Aust J Early Child.

[19] Tomczuk L, Stewart RE, Beidas RS, Mandell DS, Pellecchia M (2021). Who gets coached? A qualitative inquiry into community clinicians’ decisions to use caregiver coaching. Autism.

[20] Pickard K, Meza R, Drahota A, Brikho B (2018). They’re doing what? A brief paper on service use and attitudes in ASD community-based agencies. J Ment Health Res Intellect Disabil.

[21] Steinbrenner JR, Hume K, Odom SL, Morin KL, Nowell SW, Tomaszewski B, Szendrey S, McIntyre NS, Yücesoy-Özkan S, Savage MN (2020). Evidence-based practices for children, youth, and young adults with Autism. The University of North Carolina at Chapel Hill, Frank Porter Graham Child Development Institute, National Clearinghouse on Autism Evidence and Practice Review Team.

[22] Ingersoll B, Dvortcsak A. Teaching social communication to children with autism: a practitioner’s guide to parent training and a manual for parents. New York: Guilford Press; 2010.

[23] Minjarez MB, Karp EA, Stahmer AC, Brookman-Frazee L, Bruinsma Y, Minjarez MB, Schreibman L, Stahmer AC (2020). Empowering parents through parent training and coaching. Naturalistic developmental behavioral interventions for autism spectrum disorder.

[24] Ingersoll B, Wainer AL, Berger NI, Pickard KE, Bonter N (2016). Comparison of a self-directed and therapist-assisted telehealth parent-mediated intervention for children with ASD: a pilot RCT. J Autism Dev Disord.

[25] Akhani A, Dehghani M, Gharraee B, Shooshtari MH (2021). Parent training intervention for autism symptoms, functional emotional development, and parental stress in children with autism disorder: a randomized clinical trial. Asian J Psychiatr.

[26] Peters MDJ, Godfrey CM, Khalil H, McInerney P, Parker D, Soares CB (2015). Guidance for conducting systematic scoping reviews. JBI Evidence Implementation.

[27] Tricco AC, Lillie E, Zarin W, O’Brien KK, Colquhoun H, Levac D, Moher D, Peters MDJ, Horsley T, Weeks L, Hempel S, Akl EA, Chang C, McGowan J, Stewart L, Hartling L, Aldcroft A, Wilson MG, Garritty C, Straus SE (2018). PRISMA Extension for Scoping Reviews (PRISMA-ScR): checklist and explanation. Ann Intern Med.

[28] Damschroder LJ, Aron DC, Keith RE, Kirsh SR, Alexander JA, Lowery JC (2009). Fostering implementation of health services research findings into practice: a consolidated framework for advancing implementation science. Implement Sci.

[29] Shea CM, Jacobs SR, Esserman DA, Bruce K, Weinger BJ (2014). Organizational readiness for implementing change: a psychometric assessment of a new measure. Implementation Sci.

[30] Helfrich CD, Li YF, Sharp ND, Sales AE (2009). Organizational readiness to change assessment (ORCA): development of an instrument based on the Promoting Action on Research in Health Services (PARIHS) framework. Implement Sci.

[31] Lehman WEK, Greener JM, Simpson DD (2002). Assessing organizational readiness for change. J Subst Abuse Treat.

[32] Weiner BJ, Lewis CC, Stanick C, Powell BJ, Dorsey CN, Clary AS (2017). Psychometric assessment of three newly developed implementation outcome measures. Implement Sci.

[33] Lyon AR, Coifman J, Cook H, McRee E, Liu FF, Ludwig K, Dorsey S, Koerner K, Munson SA, McCauley E (2021). The Cognitive Walkthrough for Implementation Strategies (CWIS): a pragmatic method for assessing implementation strategy usability. Implement Sci Commun.

[34] Braun V, Clarke V (2006). Using thematic analysis in psychology. Qual Res Psychol.

[35] O’Brien BC, Harris IB, Beckman TJ, Reed DA, Cook DA (2014). Standards for reporting qualitative research: a synthesis of recommendations. Acad Med.

[36] Guetterman TC, Fetters MD, Creswell JW (2015). Integrating quantitative and qualitative results in health science mixed methods research through joint displays. Ann Fam Med.

[37] Pearson N, Naylor PJ, Ashe MC, Fernandez, M, Lin Yoong S, Wolfenden, L. Guidance for conducting feasibility and pilot studies for implementation trials. Pilot Feasibility Stud 6. 202010.1186/s40814-020-00634-w 10.1186/s40814-020-00634-w PMC7603668 33292770

[38] Pinnock H, Barwick M, Carpenter CR, StaRI Group (2017). Standards for Reporting Implementation Studies (StaRI): explanation and elaboration document. BMJ Open.

[39] Brownson RC, Shelton RC, Geng EH, Glasgow RE (2022). Revisiting concepts of evidence in implementation science. Implementation Sci.

